# Antivenomic approach of different *Crotalus durissus collilineatus* venoms

**DOI:** 10.1186/s40409-018-0169-4

**Published:** 2018-11-26

**Authors:** Isadora Sousa de Oliveira, Manuela Berto Pucca, Suely Vilela Sampaio, Eliane Candiani Arantes

**Affiliations:** 1 Department of Physics and Chemistry, School of Pharmaceutical Sciences of Ribeirão Preto, University of São Paulo, Av. do Café s/n, Monte Alegre, Ribeirão Preto, SP 14040-903 Brazil; 2 Medical School, Federal University of Roraima, Boa Vista, RR Brazil; 3 Department of Clinical Analysis, Toxicology and Food Science, School of Pharmaceutical Sciences of Ribeirão Preto, University of São Paulo, Ribeirão Preto, SP Brazil

**Keywords:** Snake venoms, *Crotalus durissus collilineatus*, Antivenomic, Crotalic antivenom, Individual variation

## Abstract

**Background:**

Our group has previously performed a proteomic study verifying that individual variations can occur among *Crotalus durissus collilineatus* venoms. These variations may lead to differences in venom toxicity and may result in lack of neutralization of some components by antivenom. In this way, this study aimed to evaluate the Brazilian anticrotalic serum capacity in recognizing twenty-two *Crotalus durissus collilineatus* venoms, as well as their fractions.

**Methods:**

The indirect enzyme-linked immunosorbent assay (ELISA) was chosen to evaluate the efficacy of heterologous anticrotalic serum produced by *Instituto Butantan* (Brazil) in recognizing the twenty-two *Crotalus durissus collilineatus* venoms and the pool of them. Moreover, the venom pool was fractionated using reversed-phase fast protein liquid chromatography (RP-FPLC) and the obtained fractions were analyzed concerning antivenom recognition.

**Results:**

Evaluation of venom variability by ELISA showed that all venom samples were recognized by the Brazilian anticrotalic antivenom. However, some particular venom fractions were poorly recognized.

**Conclusion:**

This study demonstrated that the Brazilian anticrotalic serum recognizes all the different twenty-two venoms of *C. d. collilineatus* and their fractions, although in a quantitatively different way, which may impact the effectiveness of the antivenom therapy. These results confirm the need to use a pool of venoms with the greatest possible variability in the preparation of antivenoms, in order to improve their effectiveness.

## Background

Snakebite envenoming in tropical regions is considered a serious public health problem due to its frequency and morbidity/mortality ratio, being a neglected condition belonging to the Neglected Tropical Diseases (NTD) list by World Health Organization (WHO) [1–5]. This kind of problem mainly affects rural workers, especially men and children from poor and developing countries [4, 6, 7]. Based on Brazilian epidemiological data (*Sistema de Informações de Agravos de Notificação* - SINAN, 2018), in the last decade, the number of accidents ranges from 26,000 to 30,000 per year [8]. In respect to envenomings caused by *Crotalus* genus, these accidents varies from 1,700 to 2,400 registered cases per year.

The only treatment available for snakebite envenoming is antivenom (AV) serum, in other words, hyperimmune immunoglobulins obtained from animals immunized with specific venom [9]. Therefore, more than a century after Albert Calmette’s introduction of antivenom therapy in 1895, the heterologous AV is still the unique treatment to snakebite patient recovery, although other medical practices must be also considered. For example, patients with cardiac, respiratory and renal failure should receive the AV together with emergency techniques [10, 11].

In Brazil, since 1986, with the implementation of the *Programa Nacional de Controle de Acidentes Ofídicos* by *Ministério da Saúde*, extended to other venomous animals in 1988, the production of AV was standardized. Currently, it is carried out by four institutions in the country: *Instituto Vital-Brazil*, *Instituto Butantan*, *Fundação Ezequiel Dias* (FUNED) and the *Centro de Produção e Pesquisa de Imunobiológicos* (CPPI), which are distributed by the Brazilian Ministry of Health free of charge to health institutions [12].

The first step of AV production is the extraction of the venoms that compose the mixture that is used as antigen. This mixture comprises venoms from different species and/or subspecies belonging to the same genus. Antigens are inoculated into horses (immunization process), followed by an exploratory bleeding (about 15–30 days after) to investigate the specific antibodies titration. If antibodies high titles are achieved, horse bleeding is performed. Then, plasma is separated and purified of active immunoglobulins (IgGs), which can be prepared in three main conformations [13]: monovalent Fab [14], F(ab’)_2_ fragments [15, 16] and whole IgG [17, 18]. Currently in Brazil, there are five types of AV directed to snake’s envenomings: *Bothrops* AV (*B. jararaca* – 50%; *B. jararacussu* – 12.5%; *B. neuweidi* – 12.5%; *B. alternatus* – 12.5%; *B. moojeni* – 12.5%), *Crotalus* AV (*C. d. terrificus* – 50%; *C. d. collilineatus* – 50%), *Micrurus* AV (*M. corallinus* – 50%; *M. frontalis* – 50%), *Bothrops*-*Crotalus* AV (*B. jararaca* – 50%; *B. jararacussu* – 12.5%; *B. neuweidi* – 12.5%; *B. alternatus* – 12.5%; *B. moojeni* – 12.5%; *C. d. terrificus* – 50%; *C. d. collilineatus* – 50%) and *Bothrops*-*Lachesis* AV (*B. jararaca* – 50%; *B. jararacussu* – 12.5%; *B. neuweidi* – 12.5%; *B. alternatus* – 12.5%; *B. moojeni* – 12.5%; *L. muta* – 100%) [12].

Although the antivenom therapy has proven its efficacy in preventing deaths by snakebites, AV production has not been significantly modified during a century, needing some improvements regarding quality parameters [13]. Knowing that components of venomous animals may vary according to species, genus, habitat, age, diet, among other factors, it is difficult to select venoms that will compose the antigens to be used in the AV production [19–23]. Thus, individual variations studies are necessary for a better understanding of envenoming, besides assisting in the development of a more effective AV. If the venom mixture used in the immunization does not present all toxins relevant to the envenoming, the AV may be less efficient, which will result in non-neutralized toxic effects and the use of additional doses of AV. This may lead to the manifestation of therapy side effects, such as anaphylactic reactions (non-IgE and IgE-mediated) and serum disease [24, 25].

AV potential can be evaluated through techniques named “Antivenomic”, that is the identification of venom components by proteomic techniques, which have their epitopes recognized by AV [26]. So far, there are four different ways to perform antivenomics [27]: (i) Venom and AV are mixed and the components that are recognized by the AV are precipitated. The supernatant is evaluated by reversed-phase high performance liquid chromatography (RP-HPLC) considering the chromatographic profile of the whole venom as a control [28]; (ii) AV is attached to an affinity matrix used for chromatography, which components eluted first or that do not interact with the matrix (i.e. are not recognized by AV), and those that bind in the matrix and are eluted later with a pH change, are analyzed by RP-HPLC and compared to the chromatographic profile of the whole venom [29]; (iii) Venoms are separated by two-dimensional electrophoresis, transferred to immunoblotting membranes, which are incubated with AV and the binding of antibodies in protein spots is checked [30]. Similarly, fractions obtained in RP-HPLC from venoms are analyzed by SDS-PAGE, which are also transferred to immunoblotting membranes and the process with AV is the same [28]; (iv) The last technique consists of the combination of HPLC and enzyme-linked immunosorbent assay (ELISA), in order that RP-HPLC fractions eluted are applied into microplates, sensitizing them, and the ELISA method is performed using AV as the primary antibody [31].

Based on that, this study reports the recognition potential of the antivenom produced by *Instituto Butantan* (Brazil) against twenty-two *C. d. collilineatus* venoms and their fractions through an antivenomic approach combining liquid chromatography and ELISA methods, since intraspecific venom variations may affect the efficacy of the antidote.

## Methods

### Snake venoms and antivenom

Twenty-two adult specimens of *C. d. collilineatus* were collected in the surrounding area of Catalão – GO (18° 10′ 12” S, 47° 56′ 31” W) and kept in the Serpentarium (Universidade de São Paulo, Ribeirão Preto, SP, Brazil), accredited by Brazilian Institute of Environment and Renewable Natural Resources (IBAMA), under register number 1506748, for scientific purposes. The venoms were extracted and dried under vaccum at room temperature for 6 h and stored at − 20 °C until use. The presence of crotamine in each venom was determined using mass spectrometry and N-terminal sequencing (data already published, see Oliveira et al., 2018 [32]). Pooled venom was prepared by mixing equal amount of each venom.

The heterologous antivenom against *Crotalus* venom was kindly provided by *Unidade de Farmácia do Hospital das Clínicas de Ribeirão Preto* (anticrotalic serum, lot 1208195, *Instituto Butantan*, São Paulo, Brazil).

### Venom fractionation

The venom was fractionated using a method previously described by Calvete et al. and our group [32, 33]. Briefly, the pooled venom (22 mg, 1 mg of each venom) was dispersed in 1.1 mL of 0.1% TFA (solution A) and 1% formic acid, centrifuged at 13,000 × *g* for 10 min at 4 °C. Fractionation was performed on a C18 column (250 × 10 mm, 5 μm particles, 300 Å, Phenomenex, Torrence, CA, USA) coupled to Fast Protein Liquid Chromatography (FPLC) system (Äkta Purifier UPC 900, GE Healthcare, Uppsala, Sweden). Protein elution was monitored by absorbance at 214 nm and eluted fractions were collected, frozen and lyophilized for further analysis.

### Protein quantification

Protein quantification of venoms was performed by 280/205 nm absorption method [34], while protein quantification of RP-FPLC fraction was performed in NanoDrop 2000 Microvolume Spectrophotometer (Thermo Fisher Scientific, Waltham, Massachusetts, USA), using preconfigured method Protein A_280_.

### Immunoreactivity of antivenom against venoms and their components using ELISA

An indirect ELISA was performed. 96-well microplates (Kasvi, Curitiba, PR, Brazil) were sensitized with protein (venom or fraction – 2 μg) in 0.05 M carbonate/bicarbonate buffer, pH 9.6 (100 μL/well) and incubated for 16 h at 4 °C. As positive control, the wells were sensitized with anticrotalic serum (1:1000 in 0.05 M carbonate/bicarbonate buffer, pH 9.6) and, as negative control, no sensitized wells were used. Plates were washed three times with phosphate buffered saline (PBS) pH 7.2, blocked by adding 250 μL of PBS containing 2% (*w*/*v*) powdered milk (Molico, São Paulo, SP, Brazil) (MPBS) and incubated for 2 h at 37 °C. The plates were then washed three times with PBS-0.05% Tween (PBS-T) and three times with PBS. The plates were incubated again for 1 h at 37 °C with anticrotalic serum (1:100 in 1% MPBS). The plates were washed three times with PBS-T and three times with PBS. After that, plates were incubated with 100 μL of anti-horse polyclonal antibodies conjugated with peroxidase (IgG-HRP, A6917, Sigma-Aldrich, St. Louis, MO, USA) diluted 1:3000 in 1% MPBS. After one hour of incubation at room temperature, the plates were then washed three times with PBS-T and three times with PBS. 100 μL of OPD-H_2_O_2_ (SIGMAFAST OPD tablet, SLBM4528V, Sigma-Aldrich, St. Louis, MO, USA, diluted according to the manufacturer’s instructions) were added to each well. Finally, the plates were incubated for 15 min at room temperature for the development of color (in the dark) and the reaction was interrupted with 50 μL of 1 M H_2_SO_4_ (Merck, São Paulo, SP, Brazil). Absorbance reading was performed at 490 nm on a 96-well plate reader (Sunrise-basic Tecan, Männedorf, Switzerland). The assay was carried out in quadruplicate and the results were analyzed by GraphPad Prism 5 software (La Jolla, CA, USA), using one-way ANOVA, followed by Tukey’s post-hoc test.

### Densitometry of SDS-PAGE profile of fractions

The densitometric analysis of the Tris-Tricine-SDS-PAGE (16.5%) profile of RP-FPLC fraction 0 and SDS-PAGE (12.5%) profile of RP-FPLC fractions 19, 21, 22, 35, 36, 37, 39 and 42 [32] was performed using a gel documentation system Gel Doc™ EZ System (Bio-Rad Laboratories, Inc., California, USA) and the accompanying software Image Lab™, version 5.2.1 (Bio-Rad Laboratories, Inc., California, USA).

## Results

**Fig. 1:**
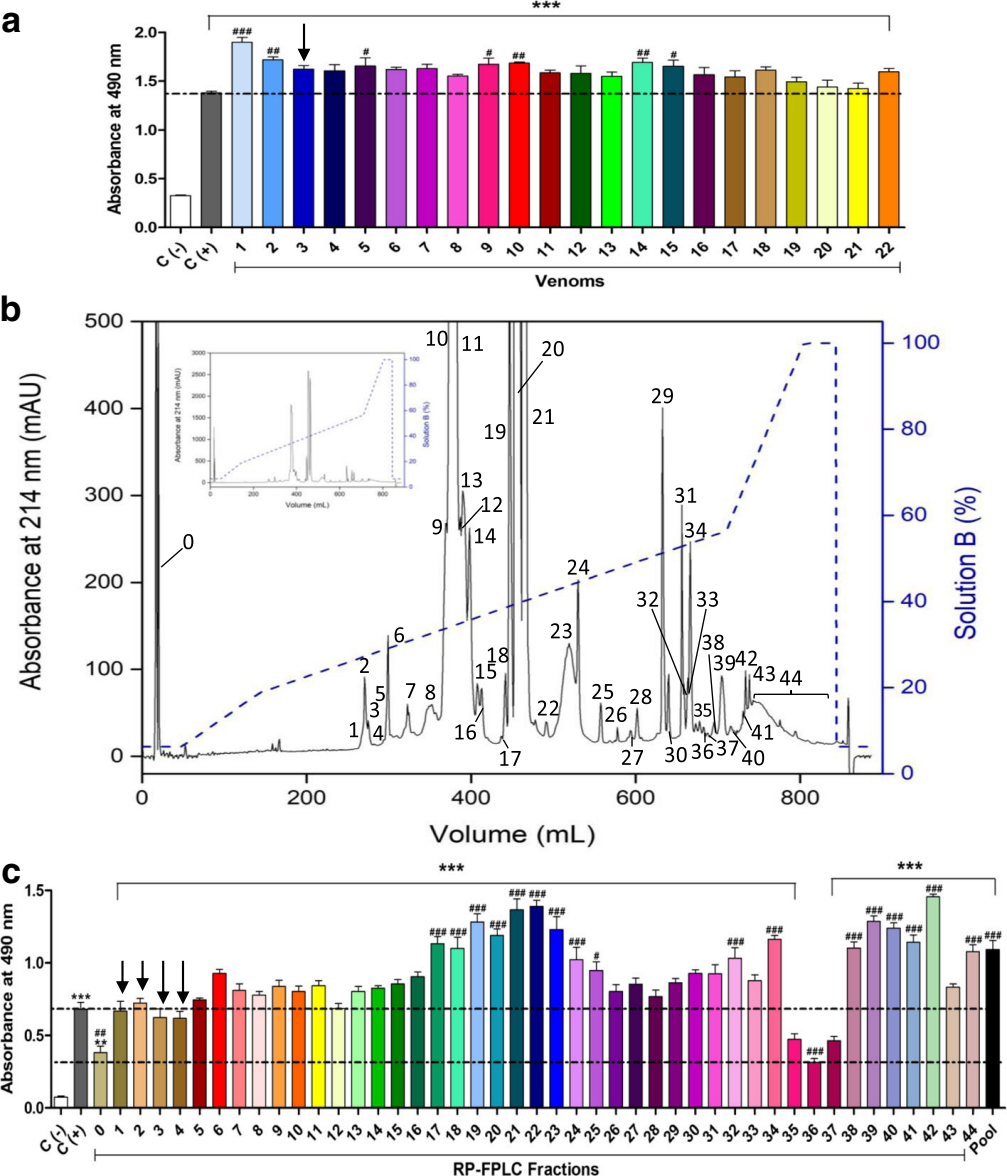
Antivenom recognition of *C. d. collilineatus* venoms and fractions performed by indirect enzyme-linked immunosorbent assay (ELISA) and chromatographic profiles of pooled venom. The 96-well plates were sensitized with 2 μg of **(a)** venoms (1–22) and **(c)** RP-FPLC fractions (0–44) diluted to 100 μL with carbonate-bicarbonate buffer (pH 9.6). The commercial anticrotalic serum (1,100) from *Instituto Butantan* was used to evaluate its capacity to recognize the venoms and their fractions using antihorse polyclonal antibodies peroxidase-labeled (1,3000) as secondary antibody. Positive control (C+): wells sensitized with anticrotalic antivenom (represented by horizontal dashed lines). Negative control (C-): non-sensitized wells. Absorbance reading was performed at 490 nm. Data are presented as mean ± SD, which were analyzed by ANOVA and Tukey’s multiple comparison test (quadruplicate assay). *p < 0.05, **p < 0.01 and ***p < 0.001 compared to C-; ^#^*p* < 0.05, ^##^*p* < 0.01 and ^###^*p* < 0.001 compared to C+. The arrow indicates crotamine-positive venom and fractions. **(b)** RP-FPLC of *C. d. collilineatus* pooled venom (22 mg) on a C18 column was carried out in a segmented concentration gradient from 6.3 to 100% of solution B (80% ACN in 0.1% TFA, represented by the blue dashed line) at a flow rate of 5 mL/min. Inset panel – whole chromatographic profile without magnification

The commercial anticrotalic serum produced by *Instituto Butantan* (Brazil) was able to recognize all the twenty-two tested venoms. All tests showed high absorbance values (≥ 1.3 at 490 nm), which indicates that the AV presents high concentration of specific antibodies and/or antibodies with high affinity against the venom components (Fig. 1a).

**Fig. 2:**
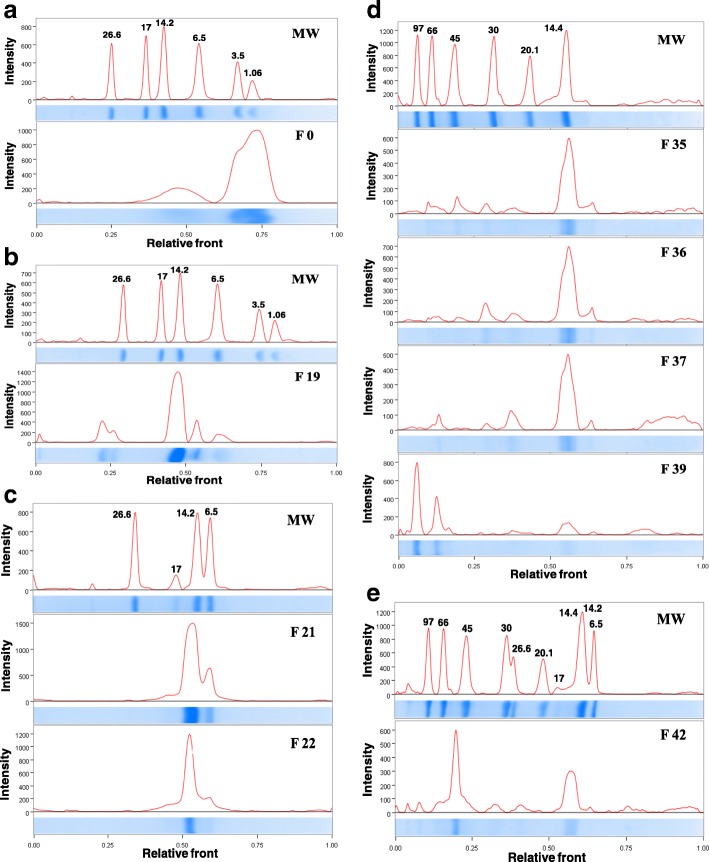
Densitometric analyses of the SDS-PAGE profiles of RP-FPLC fractions. **(a)** Fraction 0, **(b)** Fraction 19, **(c)** Fractions 21 and 22, **(d)** Fractions 35, 36, 37 and 39 and **(e)** Fraction 42. MW: molecular weight. Absorbance at 302 nm and the graphic created by software Image Lab™, version 5.2.1 (Bio-Rad Laboratories, Inc., California, USA)

Fractionation of the venom pool resulted in 44 fractions (Fig. 1b). The AV was also able to recognize all the fractions eluted from RP-FPLC, but the results obtained were very different among the different fractions tested, although the same mass of each fraction (2 μg) was used to sensitize the wells of the plate. The fractions 0, 35, 36 and 37 showed the lowest absorbance signals and fractions 19, 21, 22, 39 and 42 the highest absorbances (Fig. 1c). The densitometric analyses of the SDS-PAGE profiles of RP-FPLC fractions 0, 19, 21, 22, 35, 36, 37, 39 and 42 were shown in Fig. 2.

## Discussion

ELISA method has been shown to be a specific, cheap, simple, sensitive and fast-performing assay for detecting toxins and snake venoms [35, 36]. In the last decades, this methodology has been used for several purposes, such as determining the potency of AVs [37, 38] and detecting levels of antigens and antibodies in body fluids of patients who are victims of envenoming [39]. Furthermore, antivenomic studies may provide information of which components of a venom can be recognized by AV. Here it is important to mention that to have an efficient neutralizing effect the AV does not need to recognize all the venom components (indeed many components can be non-toxic to humans) [40, 41]. However, based on the fact that many venom’s compounds are still unknown or do not have their effects determined, the AV producers use the whole venom to immunize animals aiming to produce specific antibodies against the most components they can. Moreover, antivenomic can indirectly show the relative immunogenicity of the venom components for immunized animals [27].

Considering that the AV recognizes all the tested venoms with high absorbance, we can indirectly infer that the Brazilian anticrotalic venom presents high concentration of specific antibodies and/or high affinity antibodies against *C. d. collilineatus* venoms. Indeed, many studies demonstrated controversial ideas in this respect [42, 43]. However, ELISA appears to correlate well with both parameters: antibodies concentration and affinity.

On the other hand, concerning venom fractions (Fig. 1b), the AV recognition varies significantly (Fig. 1c). We considered that this variation can be a result of two different factors. (1) Low immunogenicity of some toxins, which hinders the production of specific and high affinity antibodies by the horses. (2) Low abundance of some components in the venom. The fractions 0, 35, 36 and 37 were poorly recognized by the AV (mean absorbance at 490 nm of 0.38, 0.47, 0.31 and 0.46, respectively), compared to fractions 21 and 22 (mean absorbance at 490 nm of 1.37 and 1.39, respectively). According to our previous proteomic analysis [32], fraction 0 (do not interact with the column) represents 2.38% of the soluble venom. It presents only small peptides (< 3 kDa; Fig. 2a) and, probably, non-protein components, which explain its low immunogenicity. Fractions 35, 36 and 37 correspond to 0.24, 0.20 and 0.15% of the venom, respectively [32]. Therefore, they are in very small proportions in the venom, justifying the low concentration of antibodies in AV. These fractions are composed by a complex mixture of toxins, mainly α (18,141 Da) and β (17,403 Da) subunits of convulxin (Figs. 2d), probably aggregated with small amounts of other toxins, such as serine proteases, 5′-nucleotidase, metalloprotease, glutathione peroxidase, carboxypeptidase, L-amino acid oxidase [32].

Fractions 19 to 22 (Fig 2b and c) are constituted by the different PLA_2_ proteoforms (crotoxin B, catalytically active) and are present in large proportions in the pooled *C. d. collilineatus* venom, corresponding to approximately 44% of the soluble venom [32], explaining its effective recognition by AV. On the other hand, fractions 39 (0.77%) and 42 (0.40%) are present in small amounts in the soluble venom, but were also very well recognized by AV (Fig. 1c). This may be justified by the fact that both are composed by toxins of high molecular masses and, consequently, with greater immunogenic potential. Nine different molecules were identified in fraction 39 [32], but those that are present in larger proportions (Fig. 2d) are phosphodiesterase (MM ~ 96.4 kDa) and 5′-nucleotidase (MM ~ 64 kDa). Fraction 42 also has a great diversity of molecules (13 toxins) [32], among them a metalloprotease (MM ~ 46 kDa), which is the toxin in the highest proportion in this fraction (Fig. 2e).

Interestingly, the unique crotamine-positive venom (number 3) was efficiently recognized by AV (Fig. 1a), but the crotamine-positive fraction (mainly fraction 2) showed only medium AV recognition (absorbance 0.73 at 490 nm) (Fig. 1c). Probably, crotamine is present in a low proportion in the mixture of venoms used for immunizing horses or it may be slightly immunogenic because of its low molecular weight (4,890 Da) [44]. It is able to cause myotoxicity, acting on muscle fibers, depolarizing cells [45] and leads to extension and induction of paralysis of the hind paws of mice, since it acts by blocking potassium channels [46].

Boldrini-França et al. reported that crotamine was not recognized by the anticrotalic sera produced by *Instituto Vital-Brazil* and *Instituto Butantan* [47], which may have led to improvements in the crotalic AV production in Brazil. Due to this lack of crotamine neutralization, Teixeira-Araújo et al. established a new protocol to anticrotalic serum production by *Instituto Vital-Brazil*, using crotamine-positive and negative crotalic venom in the same proportion for horses immunization, which resulted in the recognition of crotamine by the new AV [48]. While in *Instituto Butantan*, according to the institution, a mixture of equal amounts of *C. d. terrificus* and *C. d. collilineatus* venoms collected in different regions of Brazil is used to the antivenom production. It is worth to mention that the institution uses venoms from crotamine-negative and positive individuals and, as far as possible, from both male and female animals (*Instituto Butantan*). Indeed, the Brazilian Health Regulatory Agency (*Agência Nacional de Vigilância Sanitária* – ANVISA) recommends the use of crotamine-positive venoms for animal immunization for AV production. However, there is no standardization of crotamine percentage used in these venom mixtures. It can generate a problem, since horses can produce a weak immune response to this toxin, when immunized with low concentrations of it [48]. Therefore, our results confirm that in the last years *Instituto Butantan* has improved the quality of its antivenoms, because in the past they used to employ venoms collected in crotamine-negative regions (Southeastern and Midwestern Brazil, in the states of São Paulo, Mato Grosso and Minas Gerais) [47], as well as, the serum produced by *Instituto Vital-Brazil*, as described by Teixeira-Araujo et al. [48]. Although now AV recognizes crotamine, the low recognition of crotamine-positive fraction 2 could indicate low concentration or low affinity antibodies to crotamine in AV. Perhaps this problem can be minimized with the addition of pure crotamine in the venom mixture used for the immunization of horses, since its immunogenicity has previously been demonstrated [47, 48].

The antivenomic technique combining HPLC and ELISA used here has already been used in several other studies. Lauridsen et al. verified through this technique that South African AV was able to recognize more strongly α-neurotoxins from *Naja melanoleuca* venom, when compared to others African antivenons [31]. Laustsen et al. also showed that the African antivenoms present higher titers against high molecular mass and less toxic proteins and also against α-neurotoxins, but not as much as dendrotoxins from *Dendroaspis polylepis* venom [49].

Regarding venomous sea snakes, Laustsen et al. demonstrated that the BioCSL Sea Snake Antivenom is able to bind in neurotoxins from *Aipysurus laevis* venom, which can be effective on the treatment of this kind of envenoming [50]. Rey-Suárez et al. shown that the AV against the venom of *Micrurus nigrocinctus* is effective against the venom of *M. dumerilii* [51]. This AV was also able to recognize *M. clarki* venom [52].

Although antivenomic studies performed by ELISA presents some limitations, such as not allowing quantitative analysis, this methodology contributes considerably to toxinology field, being able to determine the antigenicity of venom components, as well as their immunoreactivity [27].

The present work emphasizes the importance of antivenomic studies, since the venom can suffer variations due to several factors, which can change the protein expression by individual, thus, there are differences in venom composition. These venom variations may difficult the treatment of victim, due to a serum that does not recognize all the components of venom, not neutralizing them, thus reducing its effectiveness.

## Conclusion

Snakebite envenomings are still neglected occupational diseases, which are in dire need of improved treatments. Although presenting some differences, the commercial antivenom produced by *Instituto Butantan* was able to recognize all the twenty-two tested venoms and their fractions, indicating that the Brazilian anticrotalic antivenom is effective in the treatment of envenomings caused by snakes of this species. Studies with antivenomic approach may reveal which components of the venom are or are not recognized by a particular AV, contributing to improve its efficacy. It makes antivenomic studies increasingly important.

## Abbreviations

ANOVA: Analysis of variance
ANVISA: Brazilian Health Regulatory Agency (*Agência Nacional de Vigilância Sanitária*)
AV: Antivenom
CPPI: *Centro de Produção e Pesquisa de Imunobiológicos*
ELISA: Enzyme-linked immunosorbent assay
FPLC: Fast protein liquid chromatography
FUNED: *Fundação Ezequiel Dias*
GO: Goiás
HPLC: High performance liquid chromatography
IBAMA: Brazilian Institute of Environment and Renewable Natural Resources
IgG-HRP: Anti-horse polyclonal antibodies conjugated with peroxidase
MM: Molecular mass
NTD: Neglected Tropical Diseases
PAGE: Polyacrylamide gel electrophoresis
PBS: Phosphate buffered saline
RP-HPLC: Reversed-phase high performance liquid chromatography
SDS: Sodium dodecyl sulfate
SINAN: *Sistema de Informações de Agravos de Notificação*
WHO: World Health Organization
